# Assessment of seasonal variation of diet composition in rodents using DNA barcoding and Real-Time PCR

**DOI:** 10.1038/s41598-019-50676-1

**Published:** 2019-10-01

**Authors:** Filippo Dell’Agnello, Chiara Natali, Sandro Bertolino, Lorenzo Fattorini, Ettore Fedele, Bruno Foggi, Matilde Martini, Caterina Pisani, Francesco Riga, Antonio Sgarlata, Claudio Ciofi, Marco Zaccaroni

**Affiliations:** 1 Department of Biology, University of Florence, Via Madonna del Piano 6, 50019 Sesto Fiorentino (FI), Italy; 2 Department of Life Sciences and Systems Biology, University of Turin, Via Accademia Albertina 13, 10123 Torino, Italy; 3 Department of Political Economy and Statistics, University of Siena, Piazza San Francesco 7-8, 53100 Siena, Italy; 4 Department of Wildlife, Institute for Environmental Protection and Research, Via Brancati 48, 00144 Rome, Italy

**Keywords:** PCR-based techniques, Behavioural ecology

## Abstract

The study of animal diet and feeding behaviour is a fundamental tool for the illustration of the ecological role of species in the ecosystem. However, size and quality of food intake samples make it hard for researchers to describe the diet composition of many small species. In our study, we exploited genomic tools for the analysis of the diet composition of the Savi’s pine vole (*Microtus savii*) using DNA barcoding and qPCR techniques for the identification of ingested plant species retrieved from stomach contents. In contrast with previous studies, we found that, despite being a fossorial species, the Savi’s pine vole is a selective feeder that undergoes intense superficial activity in search for food. In addition, our study shows that with a *a priori* knowledge of the candidate plant species included in animal diet, qPCR is a powerful tool to assess presence/absence, frequency of occurrence and electivity of ingested species. We conclude that this approach offers new opportunities to implement the analysis of food selection in small animals, thereby revealing a detailed picture of plant-animal interactions.

## Introduction

The study of animal feeding ecology is key to the understanding of the tight network of feeding relationships between species in ecological communities and of the dynamics of energy flow within ecosystems. Food web chains illustrate the importance of each species in maintaining communities’ integrity and explain how one species can affect the growth rate of other species populations^1^. Despite their restrained body size, herbivorous and granivorous rodents, occurring in great numbers worldwide, can significantly alter the species composition of the plant communities on which they feed, triggering important cascading effects through the trophic levels. For this reason, rodents are considered keystone species, capable of shaping the structure and function of ecosystems, making their diet and feeding behavior subject to ecological research^2,3^. However, diet composition analysis of small mammals has perplexed researchers for decades because the small-sized food items found in stomach contents and fecal samples are challenging to identify and measure.

With some 1800 species, rodents are a central focus of ecological research^4^ due to the significant impacts they have on crops and agroecosystems worldwide^5^. Despite being often described as pests, rodents are also keystone species that contribute to the maintenance of ecosystem stability by promoting such processes as pollination and seed dispersal^6–8^, carbon and nitrogen cycling^9,10^ and soil aeration via burrowing and tunneling activities^10,11^.

Conventionally, the assessment of animal diets has been conducted through the observation of foraging behaviour or via anatomical and histological characterization of stomach contents, regurgitated pellets and fecal remains (e.g.^12–14^). These studies, however, provide a partial picture of food intake because only broad food categories were determined, often biased towards the more easily identifiable items. Researchers have attempted to obtain a better picture of feeding habits using an array of different methods. These methods include enzyme electrophoresis^15^ and immunological approaches using antibodies to detect antigen binding sites^16^, biochemical methods to quantify the composition of food remains using near-infrared reflectance spectroscopy (e.g.^17,18^), stable isotope analysis (e.g.^19–22^), assessment of differences in alkalene composition of cuticular wax among plant taxa^23^ and molecular methods using separation of DNA of food by electrophoresis using either temperature or chemical gradients^24,25^. More recently, there has been a significant increase in dietary studies using molecular genetic approaches. Polymerase chain reaction (PCR) and high-throughput sequencing (HTS) allow a more thorough identification of ingested items^26,27^. In particular, a combination of PCR amplification and HTS (pyrosequencing and sequencing by synthesis) has been used to assess the diet composition of several herbivorous species (e.g.^28–34^).

It makes intuitive sense that the relative amount of food items consumed by a species is mirrored by the amount of DNA recovered, and ultimately the number of DNA sequences assigned to each item. However, obtaining quantitative data from amplicon sequencing is not as straightforward^26,27,35^. The amount of chloroplast DNA may vary with tissue cell density, DNA from different types of tissue can be degraded differentially during digestion, and of course, end-point PCR amplification efficiency can vary considerably between reactions. Sequencing data validation can be obtained by setting up feeding trials^35–37^. Alternative amplification techniques such as real-time, quantitative PCR represents a valid alternative to HTS when quantitative data are expected^38–42^. Quantitative PCR (qPCR) can provide similar datasets to HTS, while being more sensitive particularly to low template amounts^43,44^.

Although molecular genetics provide powerful tool for dietary analysis, studies need to be carefully designed. Many factors need to be considered. Indeed, samples from gut content and faeces often contain highly degraded DNA, so that only short fragment sequences can be readily amplified and sequenced^45^. PCR needs to target a DNA region (DNA barcode) which is well represented by genomic databases, and PCR primers must be designed to attach to conserved regions flanking the target sequence in order to minimise the risk of amplifying non-target and non-informative loci^26,27^ (and references therein). While many different nuclear and organellar DNA sequences were initially considered (e.g.^46,47^), the standardized DNA barcodes for plants are mainly identified in the plastid large subunit of ribulose 1,5-bisphosphate carboxylase/oxygenase gene (*rcb*L) and the maturase K (mat *K*) gene^48,49^. The chloroplast *trn*L intron, and in particular short regions of complimentary sequences that form stem-loop structures were also used to design highly conserved primers with robust amplification protocols^29,50,51^. However, some of these structures are hypervariable in size bearing multiple repeat motifs, which may hinder sequence alignment^52^. Moreover, both long and short fragments of the *trn*L intron appear to have a relatively low resolution. The P6 loop, for instance, is not suitable for the identification of the plant species belonging to the genus *Prunus*^50^, a particularly relevant issue for our study^29,53^.

We used DNA barcoding and qPCR techniques to assess the diet composition of a subterranean rodent species and Italian near-endemism, the Savi’s pine vole (*Microtus savii*). The Savi’s pine vole occurs in grasslands, ecotonal areas, fallow fields, along banks and ditches^54^. It is often considered a pest to farmland and orchards due to the significant damage it can cause on crops and fruit trees^55,56^. Despite its widespread occurrence, the diet of the Savi’s pine vole is poorly understood and information on its food preferences is virtually absent. Anecdotal observations suggest that the diet of Savi’s pine vole mostly consists of annual and perennial herbaceous plants, particularly *Graminaceae*, *Leguminosae*, *Chenopodiaceae* and *Compositae*, consumed within a short distance from the burrow exit holes. Electivity for *Rosaceae*, which include several economically important fruit trees, has yet to be determined.

Our approach was based on a priori knowledge of the plant species available within the foraging range of pine voles. Plants were sampled and identified using dichotomous keys. We used the *rbc*L gene as DNA barcode for species identification for both its relatively higher resolution power (e.g.^57^) and the relatively higher number of barcodes available in the Barcode for Life Data (BOLD) system for our dataset. Species-specific Taqman assays were then designed for qPCR amplification of DNA extracted from voles’ stomachs. In contrast to end-point, semi-quantitative PCR, qPCR allows the accumulation of amplified products to be detected and measured as the reaction progresses, during the exponential phase of amplification. Beside assessing presence or absence of a plant species, the starting template copy number can be determined with accuracy and high sensitivity over a wide range of DNA samples.

The Savi’s pine vole diet composition was assessed to measure the consumption of plants in relation to their availability and evaluate seasonal variations in food preference. To our knowledge, this is the first study that applies qPCR to quantitatively assess the diet and species preference of a small herbivorous mammal by using targeted assays as an alternative to HTS. Moreover, an in-depth understanding of the feeding ecology of endemic species that are well adapted to human-modified landscapes is of paramount importance to define their role and effect on anthropo-ecosystems^58^.

## Materials and Methods

### Study area

This study was conducted in a 1 ha peach orchard located in an agricultural area in Emilia Romagna, northern Italy (44°21′N, 11°42′E) from November 2014 to September 2015. Average annual rainfall was 750 mm and temperatures varied between +2.6 °C and +23.7 °C. The orchard had trees between 5 and 15 years old planted in rows 4.5 m apart at a distance between 1.5 m and 3 m from each other. The area was cultivated following traditional practices and periodically treated with insecticides, fungicides and herbicides. No rodenticides were used.

### Vegetation sampling

Sampling was conducted in November 2014, January 2015, March 2015, May 2015, July 2015 and September 2015. Food availability was evaluated by sampling vegetation using the quadrat method. We established a sampling grid consisting of 2,500 2 × 2 m quadrats. Each quadrat was then partitioned into 100 20 × 20 cm sub-quadrats. We randomly selected 40 quadrats by simple random sampling with replacement and we sampled 10 out of each of the 100 sub-quadrats by random sampling without replacement. We assessed species composition and richness for each quadrat, which was then rated using percent vegetation cover. Herbaceous plants were collected and placed between two sheets of blotted paper, gently patted to absorb moisture and subsequently wrapped in folded paper. Identification of plant material was conducted by means of dichotomous keys as found in Pignatti^59^ and comparison with previously identified herbarium specimens using a microscope for observation of diagnostic features. Forty-five plant species were identified as belonging to the classes Magnolopsida and Liliopsida (Supplementary Table 1, nomenclature according to Conti *et al*.^60^). Italian Law Decree no 157 of 11 February 1992 on the management and protection of homeothermic wildlife and game report Savi’s pine vole as a non-protected species and allows culling of populations at all time and by any means. Moreover, voles were euthanized during a pest control initiative and therefore no licenses were required at the time of the experiments.

### Vole sampling

Savi’s pine voles were trapped using 70 apple-baited snap traps placed approximately 10 cm from the entrance of the vole’s burrow. Active burrows were identified by first searching the study areas for burrow entrances. These were subsequently closed with soil. Burrows were then visited after 24 hours and traps placed close to those entrances that had been re-opened by voles^61^. A total of six sampling sessions were conducted along with vegetation sampling. Each sampling session consisted of six consecutive 24-hour trapping events whereby traps were checked every eight hours. A total of 84 voles (50 males and 34 females) were trapped and sampled over 144 hours of sampling effort. Weight, sex, age class and reproductive status were determined. Stomachs were removed according to Parkinson *et al*.^62^ and stored at −18 °C to minimize DNA degradation.

### Identification of DNA barcode sequence

We searched the BOLD and Genbank sequence database for mat *K* and *rcb*L gene sequences for each of the 45 species of plants characterized during our survey that were potentially part of the diet of Savi’s pine vole. We identified a 417 bp region of the *rcb*L gene available on Genbank for 40 candidate species (Supplementary Table 1). The partial *rcb*L gene sequence was located between position 455 and 872 of the *Arabidopsis thaliana* reference *rcb*L complete gene sequence (GenBank accession: U91966.1). Species-specific segregating sites were identified by comparing barcode sequences for 30 plants species, while a genus-specific segregating site was characterized for five groups of species, with each group consisting of two species belonging to the same genus (Supplementary Table 2). The segregating sites were used as target positions to design 35 unique Taqman assays using the Custom Taqman Assay Design Tool for gene expression (Thermo Fischer Scientific).

### DNA extraction

DNA was extracted from 30 plant species for which species-specific segregating sites were identified, and from one species for each of the five groups with genus-specific segregating sites. Extractions were conducted using a protocol modified from Doyle & Dickinson^63^ by incubating 200 mg of homogenized plant sample in 1 ml lysis buffer containing 200 mM Tris-HCl, 1.4 M NaCl, 20 mM EDTA, 20 mg cetyl trimethylammonium bromide (CTAB), 0.2% 2-mercaptoethanol and 10 mg silica powder for 2 h at 65 °C. Samples were centrifuged for 15 min at 13,000 rpm. One volume chloroform:iso-amyl alcohol (24:1) was added to the supernatant. The mixture was centrifuged for 20 min at 13,000 rpm and DNA precipitated by first incubating the supernatant with 1 volume isopropanol for 30 min at −80 °C. After the second round of centrifugation, the pellet was washed with 500 μl 70% ethanol, centrifuged for 15 min at 13,000 rpm and resuspended in DNase-free water. DNA was accurately quantified with a Qubit dsDNA BR assay kit in a Qubit 4.0 fluorometer and used as a reference for quantification of DNA from stomach contents.

DNA was then extracted from plants ingested by Savi’s pine vole via incubation of the entire stomach content (average weight: 443.5 ± 44.8SE mg) in a 2 ml microcentrifuge tube with 1 ml lysis buffer containing 0.1 mM Tris-HCl, 1.4 M NaCl, 20 mM EDTA and 20 mg CTAB for 3 h at 65 °C. DNA isolation was then conducted as described in the CTAB method by Mafra *et al*.^64^. Analysis of the whole stomach content ensures that all plant species contained in the stomach are sampled for DNA extraction.

### Real time PCR assay, conditions and thermal profiles

Because of DNA degradation in stomach contents, the length of barcoding regions that can be successfully amplified by PCR is generally limited to 100–250 bp fragments (see^26^ and references therein). In our study, we used real-time qPCR for dietary analysis by designing species- and genus-specific Taqman assays. Each assay included two PCR primers and a target-specific oligonucleotide probe labelled with flourescin (FAM) reporter and non-fluorescent quencher. Forward and reverse primers were designed over a 200 bp region stretching 100 bp in the 3′-5′ direction and 100 bp in the 5′-3′ direction from the target site, respectively. Taqman DNA probes had a conjugated minor groove binding (MGB) moiety attached to the 3′ end. The conjugated MGB folds into a minor groove formed in the DNA when the terminal 5–6 bp of the probe binds to the template. This provides the probe with a higher melting temperature (*T*_m_), close to the *T*_m_ of the primers, and an increased specificity for single base mismatches at elevated hybridization temperatures, thus strengthening probe binding. PCR products ranged from 59 bp to 102 bp (Table 1).

**Table 1:** Characteristics of 35 Taqman assays designed to assess presence/absence and DNA quantity of 40 plant species in 98 Savi’s pine vole stomach contents.

Genus and species	Forward primer (5′-3′)	GC %	bp	Tm	Reverse primer (5′-3′)	GC %	bp	Tm	PCR product (bp)	Taqman probe (5′-3′)	GC %	bp	Tm
*Amaranthus retroflexus*	AACATCGAGCCCGTTGCT	56	18	50	GGAAGTAAACATGTTAGTAACAGAACCTTCT	35	31	58	102	TTTGTTATGTAGCGTATCCTTT	32	22	47
*Avena barbata*	CGTCTGGAGGATCTACGAATTCC	52	23	57	CATGAGGCGGACCTTGGAAA	55	20	54	61	CCCTGCTTATACAAAAAC	39	18	43
*Bellis perennis*	CGATGGACTTACGAGCCTTGATC	52	23	57	CCAGGAACGGGCTCGATT	61	18	53	63	AAAGGGCGGTGCTATGG	59	17	49
*Capsella bursa-pastoris*	CCGTTCCAGGAGAAGAAACTCAA	48	23	55	ACATGTTAGTAACCGAACCTTCTTCAAA	36	28	56	84	TTTATTGCGTATGTAGCTT	32	19	42
*Cardamine hirsuta*	CACATCGAGCCCGTTCCA	61	18	53	ACATGTTAGTAACCGAACCTTCTTCAAA	36	28	56	94	AATTTATTGCATATGTAGCTT	24	21	43
*Cirsium arvensis*	TTACCAGCCTTGATCGGTACAAG	48	23	55	GGTCTAAAGGGTAAGCTACATAAGCAA	41	27	57	99	TCGAGCGCGTTATTGG	56	16	46
*Cynodon dactylon*	CTATCACATCGAACCCGTTCCT	50	22	55	ACAGAACCCTCTTCAAATAGATCTAATGGA	37	30	58	87	GGGAAGACAGTCAATATATCTG	41	22	51
*Echinochloa crus-gallii*	CGTTACAAAGGACGATGCTATCACA	44	25	56	GAACCCTCTTCAAATAGGTCTAATGGAT	39	28	57	101	GTTCCTGGGGAGCCAGAT	61	18	53
*Elytrigia repens*	CTATCACATCGAGCCTGTTCCT	50	22	55	ACGGAACCCTCTTCAAATAGGTCTA	44	25	56	87	CAATTTATCTGTTATGTAGCTT	27	22	46
*Erigeron bonariensis*	TCCAAGTTGAGAGAGATAAATTGAACAAGT	33	30	56	TTTAGCGGATAACCCCAATTTAGGTTT	37	27	55	86	CCCTGTTGGGCTGTACTA	56	18	50
*Erigeron sumatrensis*													
*Geranium dissectum*	CGTCTGGAGGATCTGCGAATC	57	21	56	TCAACTTGGATGCCGTGAGG	55	20	54	74	CTGCTTATGTGAAAACTT	33	18	41
*Geranium pusillum*	ATCTTCTACCGGTACATGGACAAC	46	24	56	GATGTGATAGCAGCGTCCTTTGTA	46	24	56	82	GGCTTACTAGTTTGGATCGT	45	20	50
*Geranium rotundifolium*	TCCTCAACCCGGAGTTCCA	58	19	53	ACCGGTAGAAGATTCAGCAGCTA	48	23	55	64	CTGAGGAAGCGGGTGCCGC	74	19	60
*Hordeum bulbosum*	CAGCCAATGGATCTGTTATGTAGCT	44	25	56	CCCAAATACGTTACCCACAATGGAA	44	25	56	97	TATTTGAGGAGGGTTCCGT	47	19	49
*Lolium multiflorum*	CTTACCAGTCTTGATCGTTACAAAGGA	41	27	57	AGCTACATAACAGATCCATTGGTTGTC	41	27	57	87	ATCATATCGAGCCTGTTG	44	18	46
*Lolium perenne*													
*Malva neglecta*	GGCGATGCTACCACATTGAG	55	20	54	ACAGAACCTTCTTCAAAAAGGTCTAAGG	39	28	57	94	CTGGAGAAGAAGAACAATATA	33	21	47
*Matricaria chamomilla*	GCTGCTATGGAATTGAGCCTGTT	48	23	55	GGTCTAATGGGTAAGCTACATAAGCAA	41	27	57	72	CCTGGAGAAGAGAATCA	47	17	45
*Medicago lupulina*	GACGCTGCTACCACATCGA	58	19	53	AGGTCTAAGGGATAAGCTACATAAGCA	41	27	57	76	TTGCTGGAGAAGAGAGTCA	47	19	49
*Plantago lanceolata*	GTCCGGCTCACGGGATC	71	17	54	CTAACAGAGGACGACCATACTTGT	46	24	56	63	CAAAGTGAGAGAGATAAATT	30	20	44
*Plantago major*	CTGTTATGTAGCTTACCCTTTAGACCTTT	38	29	57	GGGCTTTGAATCCAAATACATTTCCT	38	26	55	95	TTGAAGAAGGGTCTGTTACTAAC	39	23	52
*Poa annua*	GCTATCACATTGAGCCTGTTGCT	48	23	55	ACCCTCTTCAAATAGGTCTAATGGAT	38	26	55	83	GGGAAGATAACCAATGGA	44	18	46
*Poa trivialis*	CGTCTGGAGGATCTACGAATTCC	52	23	57	GTTCAACTTATCTCTTTCAACTTGGATACC	37	30	58	90	TGCTTATGCAAAAACTTTCCAA	32	22	47
*Portulaca oleracea*	CTTACCAGTCTTGATCGTTACAAAGGA	41	27	57	GGGTAAGCTACATAACAAATATATTGATTGTCT	30	33	57	92	ATCGATGCCGTTCCTG	56	16	46
*Prunus dulcis*	TTACTAACATGTTTACTTCCATTGTAGGT	31	29	54	CGCAAATCCTCCAGACGTAGAG	55	22	57	79	TTGGGTTCAAGGCCCTGCG	63	19	55
*Prunus persica*													
*Rumex crispus*	GTGCTCTACGTTTGGAGGATTTG	48	23	55	CGGGCCTTGGAAAGTTTTCGTA	50	22	55	62	CGAATTCCTCCTGCTT	50	16	43
*Rumex conglomeratus*	TCTACGTTTGGAGGATTTGCGAAT	42	24	54	GAGGCGGGCCTTGGAA	69	16	51	62	CCTGCTTATACGAAAACT	39	18	43
*Rumex obtusifolius*													
*Senecio vulgaris*	ACGGTATCCAAGTTGAAAGAGATAAATTGA	33	30	56	CGTAGTTTTTAGCGGATAGACCCAAT	42	26	56	99	ATGGTCGTCCTCTAATGGGAT	48	21	52
*Setaria verticillata*	CGTTACAAAGGACGATGCTATCACA	44	25	56	AACCCTCTTCAAATAGGTCTAATGGATA	36	28	56	100	GTTCCTGGGGAGGCAGA	65	17	52
*Sonchus arvensis*	CCCTGCGTGCTCTACGT	65	17	52	GCGGACCTTGGAAAGTTTTAACATA	40	25	54	69	GAAGATTTACGAATCCCTA	37	19	45
*Sonchus asper*													
*Stachys arvensis*	TCGTTACAAAGGGCGATGCT	50	20	52	AGGTCTAAAGGGTAAGCTACATAACAG	41	27	57	87	CACATCGAGACCGTTCTT	50	18	48
*Taraxacum officinale*	CGTGCTCTACGTCTGGAAGATTTG	50	24	57	GCGGACCTTGGAAAGTTTTAACAT	42	24	54	64	CGAATCCCTGTTGCGT	56	16	46
*Trifolium pratense*	CCTTAGACCTTTTTGAAGAAGGTTCTGT	39	28	57	CGTAGAGCACGCAAGGC	65	17	52	91	CATGTTTACCTCTATTGTAGG	38	21	49
*Trifolium repens*	TGTAGGTAATGTATTTGGGTTCAAGGC	41	27	57	GAGGAGGACCTTGGAAAGTTTTAACA	42	26	56	98	CTACGCCTGGAAGATTT	47	19	45
*Veronica agrestis*	GGCGCTGCGGTAGCA	73	15	50	CCATCGGTCCACACAGTTGTC	57	21	56		ATGTACCAGTCGAAGATC	44	18	46
*Veronica persica*	GGCGCTGCGGTAGCA	73	15	50	CCATCGGTCCACACAGTTGT	55	20	54	59	ATCTTCAACCGGTACATGG	47	19	49

Plant species for which only a genus-specific Taqman assay was designed are reported in bold.

A total of 97 qPCRs were performed for each candidate plant species (or genus) to quantify and assess plant species presence/absence in Savi’s pine vole stomach contents. Amplification reactions included a negative control, a standard dilution series made of three replicates of each of four 10-fold serial dilutions of candidate plant species (or genus) DNA of known concentration, and DNA samples with target-specific Taqman assay. Samples also included an exogenous internal positive control (Thermo Fisher Scientific). The internal positive control (IPC) is a single-stranded, short synthetic DNA template which is added to each amplification reaction along with a pair of specific primers and a Taqman probe labelled with a VIC fluorescent reporter. The IPC was used to distinguish between true negative results and negative results caused by PCR inhibitors, incorrect assay setup, or reagent or thermocycler failure.

Real-time PCR experiments were performed in a QuantStudio 7 Flex Real-Time PCR System (Thermo Fisher Scientific) equipped with a Fast 96-well block. Amplification reactions were conducted in 20 μl total volume containing 1X Taqman Fast Advanced Master Mix (Thermo Fisher Scientific), 0.9 μM each primer, 0.25 μM Taqman probe, 0.36 μM each IPC primer, 0.1 μM IPC Taqman probe and 10 ng of IPC and sample DNA. A ROX fluorescent dye included in the Master Mix was used as an internal passive reference to normalized PCR fluorescent dye signals. We used a passive reference to correct for possible fluorescent fluctuations including well-to-well volume or light source intensity variations, minor changes in concentration, and non-PCR related fluctuations caused, for instance, by pipetting errors. Thermal-cycling profiles consisted of a denaturation step at 95 °C for 20 s, followed by 40 cycles of 1 s at 95 °C and annealing/extension of 20 s at 60 °C. Filter sets were x1-m1, x1-m2 and x4-m4 for FAM, VIC and ROX, respectively. The number of initial cycles of the PCR during which background fluorescent signal is produced (baseline) and the threshold value whereby enough amplified product has accumulated to yield a detectable fluorescent signal were set automatically by the QuantStudio Real-Time PCR software 1.0.

### Standard curve

Amplification of four 10-fold serial dilutions of DNA template of known concentration was used to generate a standard curve by plotting the log-scaled starting quantity of DNA template (N_0_) against the threshold cycle (C_T_) value obtained during amplification of each dilution. The C_T_ values of the samples of unknown concentration were compared to the standard curve to derive the quantity of starting DNA concentration. Three replicates of each dilution point in the standard curve were performed to ensure statistical significance.

Performance of Real-Time PCR reactions was evaluated by the Pearson Correlation Coefficient and the slope of the regression line of the standard curve. Assuming accurate aliquoting and no changes in the efficiency of the amplification over the range of DNA concentrations (i.e. the amount of PCR product doubles during each cycle of exponential amplification resulting in a 100% reaction efficiency), the dilution series should result in amplification curves that are evenly spaced by a number of cycles equal to the log_2_ of the dilution factor. With a 10-fold serial dilution of DNA we expected the C_T_ values of each dilution separated by approximately 3.32 cycles. Considering that the slope of the regression line with equation *y* = a*x* is the change in *y* for a unit change in *x* along the line, then a change in DNA concentration of one logarithmic unit (10-fold increase) should correspond to a 3.32 cycle decrease, and an expected slope value of -3.32. Amplification efficiency (E) was calculated from the slope of the standard curve using the equation:

$${\rm{E}}={10}^{-1/{\rm{slope}}}-1$$

derived from Rutledge & Côté^65^ by putting C_T_ on the *y*-axis and log (N_0_) on the *x*-axis.

### Statistical analysis

The mean percentage cover of each plant species and bare ground, along with associated variance, were calculated by averaging the Horvitz-Thompson estimates of percent coverage obtained from the 40 vegetation survey quadrats^66^. As we were only interested in the proportional abundance of plant species, we excluded bare ground data and re-scaled plant cover data to between 0–100. The sampling variances of the scaled estimates were calculated using the delta method (e.g.^67^).

The amount of plant DNA recovered from each stomach content by qPCR was used as a proxy for the proportion of each plant species ingested by an individual vole. As the peach, *Prunus persica*, is an arboreal species and the Savi’s pine vole is known to feed upon roots rather than aerial parts of the plant, we were not able to estimate its availability. Therefore, no analysis on food selection could be performed and the amount of *P*. *persica* DNA recovered in the stomach contents was not considered when quantifying the proportion of plant species ingested.

For each trapping session a sign test was performed to assess the selection of plant species by the Savi’s pine vole^68^. The test statistic was based on the number of animals with a percent of plant DNA in the stomach higher than the plant percent availability in the study area. Plant availability was estimated using the previously described sampling strategy and could therefore be equal to zero for some species, because either a species was not available in the study area or it was not detected in the sampled sub-quadrats. Presence of DNA in at least one stomach content for a species of plant estimated as not available in the study area highlighted that the zero estimate was due to a sampling error. Then, the availability of that species was considered greater than its proportional use also for those individuals that did not feed on that plant and therefore had a percentage of use equal to zero. For each plant species, p-values of sign tests were derived by means of the binomial probability distribution and subsequently combined in a test statistic to assess the overall null hypothesis of no plant selection by Savi’s pine voles. Statistical significance of the overall null hypothesis was determined by permuting sample observations^68^. The hypothesis of no plant selection was rejected for all six trapping sessions. The p-values of the tests performed for each plant species were used to partition the set of available plant species into preferred, avoided and proportionally used food items. Significance level of the tests for each plant species was set equal to 0.05. Analyses were performed in R^69^ using the “phuassess” package^70^, available from the Comprehensive R Archive Network (CRAN).

## Results

Table 2 displays the percentages of estimated plant cover in the study area collected during each sampling session. Approximately 50% were perennial species while the other half were annual plants. Couch grass (*Elytrigia repens*), ribwort plantain (*Plantago lanceolata*), broadleaf plantain (*Plantago major*) and common dandelion (*Taraxacum officinale*) were the dominant species, which, despite seasonal variation of vegetation cover, accounted for the majority of plants available to the Savi’s pine vole.

**Table 2:** Estimated percent of plant availability for each trapping session.

Species	Nov.	Jan.	Mar.	May	Jul.	Sep.
*Amaranthus retroflexus*	0.00	0.00	0.00	0.00	3.60	2.53
*Avena barbata*	0.00	0.00	0.00	0.00	2.96	1.90
*Bellis perennis*	1.24	1.69	2.59	1.16	1.00	0.41
*Capsella bursa pastoris*	3.04	0.24	0.17	0.38	0.13	0.00
*Cardamina hirsuta*	0.05	0.11	3.72	0.00	0.00	0.00
*Cirsium arvensis*	0.25	0.00	0.00	0.00	0.00	0.00
*Cynodon dactylon*	0.00	0.00	0.21	0.00	5.27	5.50
*Echinochloa crus-gallii*	0.50	0.00	0.16	0.06	0.00	0.34
*Elytrigia repens*	30.74	35.76	30.05	22.25	30.07	25.85
*Erigeron* sp.	2.16	1.25	0.82	3.19	1.77	1.89
*Geranium dissectum*	0.00	0.00	1.32	0.29	0.00	0.00
*Geranium pusillum*	0.83	1.42	0.46	1.83	0.57	0.04
*Geranium rotundifolium*	0.06	0.00	0.07	0.00	0.00	0.00
*Hordeum bulbosum*	0.00	0.00	0.00	3.85	0.88	0.51
*Lolium* sp.	0.00	0.00	0.00	0.06	4.60	2.37
*Malva neglecta*	5.96	3.99	0.37	1.08	0.61	0.74
*Matricaria chamomilla*	0.00	0.00	0.07	0.38	0.36	0.23
*Medicago lupulina*	0.00	0.00	1.54	0.13	0.00	0.00
*Plantago lanceolata*	7.41	8.73	9.09	14.94	11.26	12.58
*Plantago major*	3.89	1.73	4.34	3.74	8.02	12.67
*Poa annua*	0.00	0.00	4.23	7.55	4.05	3.17
*Poa trivialis*	0.00	0.00	0.03	0.00	0.00	0.00
*Portulaca oleracea*	0.00	0.00	0.00	0.26	0.51	0.59
*Rumex conglomeratus*	0.06	0.00	0.29	0.00	0.17	0.34
*Rumex crispus*	0.00	0.65	1.47	0.85	1.42	0.32
*Senecio vulgaris*	0.06	0.00	0.84	0.06	0.00	0.13
*Setaria verticillata*	0.00	0.00	0.00	0.00	1.74	9.20
*Sonchus* sp.	0.42	0.06	0.13	0.30	0.10	0.00
*Stachys arvensis*	6.29	5.15	3.10	0.11	0.00	0.00
*Taraxacum officinale*	19.77	30.97	25.14	35.57	18.25	17.21
*Trifolium pratense*	3.31	1.00	0.30	0.68	0.68	0.62
*Trifolium repens*	10.15	5.98	0.47	1.13	1.98	0.85
*Veronica persica*	3.80	1.28	9.02	0.16	0.00	0.00
Total	100.00	100.00	100.00	100.00	100.00	100.00

We trapped 20 voles in November, 15 in January, 10 in March, 15 in May, 13 in July and 11 in September. Each stomach sample contained an average of 17.5 ± 1.67SE species of plants (range: 8–24). Results of the permutation test showed that the proportion of plant species found in the voles’ stomachs did not mirror their availability in the study area (P < 0.001). The majority of plant species were found in the voles stomachs in a greater proportion with respect to their percent availability (Tables 3 and 4). In addition, we found seasonal variations in the Savi’s pine vole diet, a periodic selection of 6–8 species of plants and avoidance of between 14 and 20 other species. Seasonal selection of plants included rare species such as *Amaranthus retroflexus*, *Avena barbata*, *Lolium* sp. between November and May, and *Cardamine hirsuta* and *Geranium dissectum* from July to September. Although plant species selection by the Savi’s pine vole changed across the entire sampling period, we found that voles never fed on *T*. *officinale*, *Bellis perennis*, *Geranium pusillum*, *P*. *lanceolate*, *P*. *major*, *Setaria verticillata* and *Trifolium pratense* regardless of season and relative abundance. In March, no samples of *Setaria verticillata* were found in any of the sampling plots or SAvi’s pine vole stomachs, and it was therefore not considered in the analysis. The average proportion of the peach, *P*. *persica*, in the stomach contents was low, from 0.12% in September to 5.52% in January (Table 5). Nevertheless, this species was contained in all stomach samples during every single sampling session.

**Table 3:** Percent of Savi’s pine voles feeding on a plant species in a greater proportion than its estimated availability for each trapping session.

Species	Percent of Savi’s pine voles					
	Nov.	Jan.	Mar.	May	Jul.	Sep.
*Amaranthus retroflexus*	100.00	100.00	100.00	100.00	15.38	0.00
*Avena barbata*	100.00	100.00	100.00	100.00	0.00	0.00
*Bellis perennis*	5.00	0.00	0.00	20.00	0.00	0.00
*Capsella bursa pastoris*	0.00	6.67	0.00	6.67	0.00	100.00
*Cardamina hirsuta*	10.00	13.33	0.00	100.00	100.00	100.00
*Cirsium arvensis*	5.00	100.00	100.00	100.00	100.00	100.00
*Cynodon dactylon*	100.00	100.00	0.00	100.00	0.00	0.00
*Echinochloa crus-gallii*	10.00	100.00	0.00	33.33	100.00	0.00
*Elytrigia repens*	5.00	13.33	20.00	0.00	7.69	9.09
*Erigeron* sp.	50.00	33.33	100.00	46.67	30.77	18.18
*Geranium dissectum*	60.00	73.33	0.00	20.00	84.62	90.91
*Geranium pusillum*	0.00	0.00	0.00	0.00	0.00	0.00
*Geranium rotundifolium*	35.00	73.33	30.00	53.33	46.15	72.73
*Hordeum bulbosum*	100.00	100.00	100.00	0.00	0.00	0.00
*Lolium* sp.	100.00	100.00	100.00	46.67	0.00	0.00
*Malva neglecta*	10.00	26.67	20.00	13.33	7.69	0.00
*Matricaria chamomilla*	85.00	86.67	60.00	33.33	7.69	9.09
*Medicago lupulina*	70.00	80.00	0.00	46.67	100.00	100.00
*Plantago lanceolata*	5.00	0.00	10.00	6.67	0.00	0.00
*Plantago major*	5.00	0.00	0.00	0.00	0.00	0.00
*Poa annua*	5.00	86.67	10.00	0.00	7.69	0.00
*Poa trivialis*	40.00	93.33	70.00	60.00	84.62	54.55
*Portulaca oleracea*	100.00	100.00	100.00	13.33	76.92	90.91
*Rumex conglomeratus*	35.00	40.00	10.00	33.33	0.00	0.00
*Rumex crispus*	50.00	20.00	0.00	20.00	0.00	0.00
*Senecio vulgaris*	85.00	66.67	50.00	53.33	92.31	100.00
*Setaria verticillata*	5.00	6.67	—	6.67	0.00	0.00
*Sonchus* sp.	75.00	80.00	80.00	80.00	76.92	100.00
*Stachys arvensis*	0.00	0.00	0.00	0.00	38.46	45.45
*Taraxacum officinale*	0.00	0.00	0.00	0.00	0.00	0.00
*Trifolium pratense*	0.00	0.00	0.00	13.33	0.00	0.00
*Trifolium repens*	0.00	40.00	20.00	66.67	30.77	9.09
*Veronica persica*	5.00	0.00	0.00	20.00	46.15	54.55

**Table 4:** Food preference of Savi’s pine voles. For each trapping session, plant species are ranked according to the proportion of voles feeding on a palnt in a greater proportion than its estimated availability in the field.

Rank	November *p* = 0.00003 *n* = 20	January *p* = 0.00098 *n* = 15	March *p* = 0.02148 *n* = 10	May *p* = 0.00134 *n* = 15	July *p* = 0.00366 *n* = 13	September *p* = 0.00879 *n* = 11
1	*A*. *retroflexus* +	*A*. *retroflexus* +	*A*. *retroflexus* +	*A*. *retroflexus* +	*C*. *hirsuta* +	*C*. *hirsuta* +
2	*A*. *barbata* +	*A*. *barbata* +	*A*. *barbata* +	*A*. *barbata* +	*C*. *arvensis* +	*C*. *bursa pastoris* +
3	*C*. *dactylon* +	*C*. *arvensis* +	*C*. *arvensis* +	*C*. *hirsuta* +	*E*. *crus-gallii* +	*C*. *arvensis* +
4	*H*. *bulbosum* +	*C*. *dactylon* +	*Erigeron* sp. +	*C*. *arvensis* +	*M*. *lupolina* +	*M*. *lupolina* +
5	*Lolium* sp. +	*E*. *crus-gallii* +	*H*. *bulbosum* +	*C*. *dactylon* +	*S*. *vulgaris* +	*S*. *vulgaris* +
6	*P*. *oleracea* +	*H*. *bulbosum* +	*Lolium* sp. +	*Sonchus* sp. +	*G*. *dissectum* +	*Sonchus* sp. +
7	*M*. *chamomilla* +	*Lolium* sp. +	*P*. *oleracea* +	*T*. *repens* ▯	*P*. *trivialis* +	*G*. *dissectum* +
8	*S*. *vulgaris* +	*P*. *oleracea* +	*Sonchus* sp. ▯	*P*. *trivialis* ▯	*P*. *oleracea* ▯	*P*. *oleracea* +
9	*Sonchus* sp. +	*P*. *trivialis* +	*P*. *trivialis* ▯	*G*. *rotondifolium* ▯	*Sonchus* sp. ▯	*G*. *rotondifolium* ▯
10	*M*. *lupolina* ▯	*M*. *chamomilla* +	*M*. *chamomilla* ▯	*S*. *vulgaris* ▯	*G*. *rotondifolium* ▯	*P*. *trivialis* ▯
11	*G*. *dissectum* ▯	*P*. *annua* +	*S*. *vulgaris* ▯	*Erigeron* sp. ▯	*V*. *persica* ▯	*V*. *persica* ▯
12	*Erigeron* sp. ▯	*M*. *lupolina* +	*G*. *rotondifolium* ▯	*Lolium* sp. ▯	*S*. *arvensis* ▯	*S*. *arvensis* ▯
13	*R*. *crispus* ▯	*Sonchus* sp. +	*E*. *repens* ▯	*M*. *lupolina* ▯	*Erigeron* sp. ▯	*Erigeron* sp. ▯
14	*P*. *trivialis* ▯	*G*. *dissectum* ▯	*M*. *neglecta* ▯	*E*. *crus-gallii* ▯	*T*. *repens* ▯	*E*. *repens* −
15	*G*. *rotondifolium* ▯	*G*. *rotondifolium* ▯	*T*. *repens* ▯	*M*. *chamomilla* ▯	*A*. *retroflexus* −	*M*. *chamomilla* −
16	*R*. *conglomeratus* ▯	*S*. *vulgaris* ▯	*P*. *lanceolata* −	*R*. *conglomeratus* ▯	*E*. *repens* −	*T*. *repens* −
17	*C*. *hirsuta* −	*R*. *conglomeratus* ▯	*P*. *annua* −	*B*. *perennis* −	*M*. *chamomilla* −	*A*. *retroflexus* −
18	*E*. *crus-gallii* −	*T*. *repens* ▯	*R*. *conglomeratus* −	*G*. *dissectum* −	*M*. *neglecta* −	*A*. *barbata* −
19	*M*. *neglecta* −	*Erigeron* sp. ▯	*B*. *perennis* −	*R*. *crispus* −	*P*. *annua* −	*B*. *perennis* −
20	*B*. *perennis* −	*M*. *neglecta* ▯	*C*. *hirsuta* −	*V*. *persica* −	*A*. *barbata* −	*C*. *dactylon* −
21	*C*. *arvensis* −	*R*. *crispus* −	*C*. *bursa pastoris* −	*M*. *neglecta* −	*B*. *perennis* −	*E*. *crus-gallii* −
22	*E*. *repens* −	*C*. *hirsute* −	*C*. *dactylon* −	*P*. *oleracea* −	*C*. *bursa pastoris* −	*G*. *pusillum* −
23	*P*. *lanceolata* −	*E*. *repens* −	*E*. *crus-gallii* −	*T*. *pratense* −	*C*. *dactylon* −	*H*. *bulbosum* −
24	*P*. *major* −	*C*. *bursa pastoris* −	*G*. *dissectum* −	*C*. *bursa pastoris* −	*G*. *pusillum* −	*Lolium* sp. −
25	*P*. *annua* −	*S*. *verticillata* −	*G*. *pusillum* −	*P*. *lanceolata* −	*H*. *bulbosum* −	*M*. *neglecta* −
26	*S*. *verticillata* −	*B*. *perennis* −	*M*. *lupolina* −	*S*. *verticillata* −	*Lolium* sp. −	*P*. *lanceolata* −
27	*V*. *persica* −	*G*. *pusillum* −	*P*. *major* −	*E*. *repens* −	*P*. *lanceolata* −	*P*. *major* −
28	*C*. *bursa pastoris* −	*P*. *lanceolata* −	*R*. *crispus* −	*G*. *pusillum* −	*P*. *major* −	*P*. *annua* −
29	*G*. *pusillum* −	*P*. *major* −	*S*. *arvensis* −	*H*. *bulbosum* −	*R*. *conglomeratus* −	*R*. *conglomeratus* −
30	*S*. *arvensis* −	*S*. *arvensis* −	*T*. *officinale* −	*P*. *major* −	*R*. *crispus* −	*R*. *crispus* −
31	*T*. *officinale* −	*T*. *officinale* −	*T*. *pratense* −	*P*. *annua* −	*S*. *verticillata* −	*S*. *verticillata* −
32	*T*. *pratense* −	*T*. *pratense* −	*V*. *persica* −	*S*. *arvensis* −	*T*. *officinale* −	*T*. *officinale* −
33	*T*. *repens* −	*V*. *persica* −	−	*T*. *officinale* −	*T*. *pratense* −	*T*. *pratense* −

Samples size (*n*) and significance values of the null hypothesis of proportional vegetation use (*p*) are also reported. Symbols to the right of each plant species represent avoidance (−), proportional use (▯) or preference (+).

**Table 5:** Mean percentage of *Prunus persica* found in the stomach contents of Savi’s pine voles. *n*: sample size.

	November *n* = 20	January *n* = 20	March *n* = 10	May *n* = 15	July *n* = 13	September *n* = 11
Mean ± SE	1.90 ± 0.72	5.52 ± 1.14	1.96 ± 1.26	5.50 ± 1.68	1.64 ± 1.04	0.12 ± 0.07

## Discussion

The aim of many dietary studies is not simply to assess food item diversity but to acquire quantitative data on the relative amounts of plant species or preys ingested by an organism^26,27^ (and references therein). Our study shows that with a relatively comprehensive *a priori* knowledge of the candidate plant species an animal can possibly feed upon, the employment of qPCR can provide a good estimate of presence/absence, frequency of occurrence and electivity of each ingested species. Under this assumptions, qPCR can offer either an alternative or a complementary method to HTS, in which even a well designed dietary barcoding study is likely to provide semi-quantitative estimates of the diet of a species or frequencies of sequencing reads as a proxy of the relative abundance of dietary items^36,37,43,71^. Moreover, our results indicate that Taqman assays based on short fragments of the *rcbL* gene can perform relatively well as DNA barcodes even in significantly degraded samples such as those found in stomach contents^28,72^.

Although plant species availability in our study area was, to some extent, affected by anthropogenic disturbance (e.g. mowing and plowing) which may have altered the natural phenological cycle of plants, we found significant seasonal variability in the diet composition of the Savi’s pine vole. Indeed, our results show high levels of selectivity for some species of herbaceous plants, including *A*. *retroflexus*, *A*. *barbata*, *C*. *arvensis*, *Portulaca oleracea*, *Senecio vulg*aris and *Soncus* sp., the latter being almost always selected throughout the year. On the other hand, Savi’s pine vole appears to avoid other species such as *T*. *officinale*, *B*. *perennis*, *G*. *pusillum*, *P*. *lanceolate*, *P*. *major*, *S*. *verticillata*, and *T*. *pratense*. Although *P*. *persica* averaged only 5.5% of the overall food intake, with peaks of up to 20% in a few samples, this species was found in all stomach samples suggesting that the peach was likely consumed throughout the year. The seasonal presence of rare species of plants found in stomach contents, including *A*. *retroflexus*, *A*. *barbata*, *Lolium* sp. between November and May, and *C*. *hirsuta* and *G*. *dissectum* from July to September, suggests that the Savi’s pine vole actively selects the plant species to include in its diet. This implies intense search activities and specific behavioral and ecological patterns that may have been so far widely overlooked^73^. Based on our results, Savi’s pine vole can be indeed regarded as a pest to agroecosystems particularly at high population densities.

Interestingly, we found that despite the number of individuals varies between 2 and 32 per hectare^74^, and therefore regardless of the competition for food resources, the Savi’s pine vole feeds upon *S*. *vulgaris*, a poisonous plant which contains high concentrations of secondary toxic compounds such as pyrrolizidine alkaloids that were showed to cause liver damages and even lead to the death of a number of other herbivore species^75,76^. We suppose that the Savi’s pine vole have evolved specific physiological mechanisms that allow them to metabolise these toxins. However, because detoxification processes of chemical compounds are energy-consuming, further investigations on the factors affecting food selection would greatly contribute to the understanding of the species ecology. Particular attention should be draw on the use of pesticides in agroecosystems and the understanding of their role in mediating diet selection in the Savi’s pine vole.

Overall, our study represents an example of how qPCR can be employed for quantitative dietary analysis of herbivorous species and in particular the Savi’s pine vole, whose feeding ecology is poorly known and especially important when increases in population densities may represent a threat to agricultural ecosystems. Finally, although our study was based on the use of stomach contents, we argue that a similar approach can be used for the analysis of non-invasive samples (e.g. faeces) to minimise the disturbance and facilitating analyses of diet composition of endangered or elusive species. The choice depends on the conservation status of the species, on the degradation level of DNA samples and the respect of ethical constraints^5^.

## Supplementary information

supplementary material
