# Supplementary material for: Assessment of seasonal variation of diet composition in rodents using DNA barcoding and Real-Time PCR
Source: Sci Rep. 2019 Oct 1;9:14124. doi: 10.1038/s41598-019-50676-1 (PMC6773709; doi:10.1038/s41598-019-50676-1)
Supplement: Supplementary file 1 — supplementary material [file 41598_2019_50676_MOESM1_ESM.docx]

**Assessment of seasonal variation of diet composition in rodents using DNA barcoding and Real-Time PCR.**

Filippo Dell'Agnello^1^*, Chiara Natali^1^*, Sandro Bertolino^2^, Lorenzo Fattorini^3^, Ettore Fedele^1^, Bruno Foggi^1^, Matilde Martini^1^, Caterina Pisani^3^, Francesco Riga^4^, Antonio Sgarlata^1^, Claudio Ciofi^1^ & Marco Zaccaroni^1^

1 Department of Biology, University of Florence, Via Madonna del Piano 6, 50019 Sesto Fiorentino (FI), Italy.

2 Department of Life Sciences and Systems Biology, University of Turin, Via Accademia Albertina 13, 10123 Torino, Italy.

3 Department of Political Economy and Statistics, University of Siena, Piazza San Francesco 7-8, 53100 Siena, Italy.

4 Department of Wildlife, Institute for Environmental Protection and Research, Via Brancati 48, 00144 Rome, Italy.

*These authors contributed equally to this work

Correspondence and requests for materials should be addressed to M.Z. (email: [marco.zaccaroni@unifi.it](mailto:marco.zaccaroni@unifi.it))

**Supplementary Table 1.** Taxonomic classification of 40 plant species tested for presence and DNA quantity in Savi’s pine vole stomach contents.

| **Class** | **Order** | **Family** | **Genus and species** |
| --- | --- | --- | --- |
|  |  |  |  |
| Liliopsida | Poales | Poaceae | Avena barbata |
| Liliopsida | Poales | Poaceae | Cynodon dactylon |
| Liliopsida | Poales | Poaceae | Echinochloa crus-gallii |
| Liliopsida | Poales | Poaceae | Elytrigia repens |
| Liliopsida | Poales | Poaceae | Hordeum bulbosum |
| Liliopsida | Poales | Poaceae | Lolium multiflorum |
| Liliopsida | Poales | Poaceae | Lolium perenne |
| Liliopsida | Poales | Poaceae | Poa annua |
| Liliopsida | Poales | Poaceae | Poa trivialis |
| Liliopsida | Poales | Poaceae | Setaria verticillata |
| Magnoliopsida | Asterales | Asteraceae | Bellis perennis |
| Magnoliopsida | Asterales | Asteraceae | Erigeron bonariensis |
| Magnoliopsida | Asterales | Asteraceae | Erigeron sumatrensis |
| Magnoliopsida | Asterales | Asteraceae | Matricaria chamomilla |
| Magnoliopsida | Asterales | Asteraceae | Senecio vulgaris |
| Magnoliopsida | Asterales | Asteraceae | Sonchus arvensis |
| Magnoliopsida | Asterales | Asteraceae | Sonchus asper |
| Magnoliopsida | Asterales | Asteraceae | Taraxacum officinale |
| Magnoliopsida | Brassicales | Brassicaceae | Capsella bursa-pastoris |
| Magnoliopsida | Brassicales | Brassicaceae | Cardamine hirsuta |
| Magnoliopsida | Caryophyllales | Amaranthaceae | Amaranthus retroflexus |
| Magnoliopsida | Caryophyllales | Polygonaceae | Rumex conglomeratus |
| Magnoliopsida | Caryophyllales | Polygonaceae | Rumex crispus |
| Magnoliopsida | Caryophyllales | Polygonaceae | Rumex obtusifolius |
| Magnoliopsida | Caryophyllales | Portulacaceae | Portulaca oleracea |
| Magnoliopsida | Fabales | Fabaceae | Medicago lupulina |
| Magnoliopsida | Fabales | Fabaceae | Trifolium pratense |
| Magnoliopsida | Fabales | Fabaceae | Trifolium repens |
| Magnoliopsida | Geraniales | Geraniaceae | Geranium dissectum |
| Magnoliopsida | Geraniales | Geraniaceae | Geranium pusillum |
| Magnoliopsida | Geraniales | Geraniaceae | Geranium rotundifolium |
| Magnoliopsida | Lamiales | Lamiaceae | Stachys arvensis |
| Magnoliopsida | Lamiales | Plantaginaceae | Plantago lanceolata |
| Magnoliopsida | Lamiales | Plantaginaceae | Plantago major |
| Magnoliopsida | Lamiales | Plantaginaceae | Veronica agrestis |
| Magnoliopsida | Lamiales | Plantaginaceae | Veronica persica |
| Magnoliopsida | Malvales | Malvaceae | Malva neglecta |
| Magnoliopsida | Rosales | Rosaceae | Prunus dulcis |
| Magnoliopsida | Rosales | Rosaceae | Prunus persica |
| Magnoliopsida | Solanales | Convolvulaceae | Convolvulus arvensis |
|  |  |  |  |

**Supplementary Table 2.** Map of segregating sites for 35 Taqman probes employed to test for presence and DNA quantity of 40 plant species in Savi’s pine vole stomach contents. Taqman probes were designed to have one or a combination of more nucleotides (highlighted in grey) making each probe specific to the target species or genus DNA sequence. The number of polymorphic nucleotide sites other than the segregating ones where each probe differred from any of the other 34 template sequences are also reported. Vertical lines indicate plants for which only a genus-specific Taqman assay could be designed.

| Genus and species | Probe 5’-3’ | No of additional polymorphic sites |
| --- | --- | --- |
|  |  |  |
| Amaranthus retroflexus | TTTGTTATGTAGCGTATCCTTT | 7 |
| Avena barbata | CCCTGCTTATACAAAAAC | 6 |
| Bellis perennis | AAAGGGCGGTGCTATGG | 5 |
| Capsella bursa-pastoris | TTTATTGCGTATGTAGCTT | 7 |
| Cardamine hirsuta | AATTTATTGCATATGTAGCTT | 8 |
| Convolvulus arvensis | TCGAGCGCGTTATTGG | 6 |
| Cynodon dactylon | GGGAAGACAGTCAATATATCTG | 10 |
| Echinochloa crus-gallii | GTTCCTGGGGAGCCAGAT | 8 |
| Elytrigia repens | CAATTTATCTGTTATGTAGCTT | 6 |
| Erigeron bonariensis | CCCTGTTGGGCTGTACTA | 6 |
| Erigeron sumatrensis |  |  |
| Geranium dissectum | CTGCTTATGTGAAAACTT | 5 |
| Geranium pusillum | GGCTTACTAGTTTGGATCGT | 6 |
| Geranium rotundifolium | CTGAGGAAGCGGGTGCCGC | 4 |
| Hordeum bulbosum | TATTTGAGGAGGGTTCCGT | 4 |
| Lolium multiflorum | ATCATATCGAGCCTGTTG | 8 |
| Lolium perenne |  |  |
| Malva neglecta | CTGGAGAAGAAGAACAATATA | 10 |
| Matricaria chamomilla | CCTGGAGAAGAGAATCA | 10 |
| Medicago lupulina | TTGCTGGAGAAGAGAGTCA | 11 |
| Plantago lanceolata | CAAAGTGAGAGAGATAAATT | 5 |
| Plantago major | TTGAAGAAGGGTCTGTTACTAAC | 5 |
| Poa annua | GGGAAGATAACCAATGGA | 9 |
| Poa trivialis | TGCTTATGCAAAAACTTTCCAA | 4 |
| Portulaca oleracea | ATCGATGCCGTTCCTG | 8 |
| Prunus dulcis | TTGGGTTCAAGGCCCTGCG | 2 |
| Prunus persica |  |  |
| Rumex crispus | CGAATTCCTCCTGCTT | 4 |
| Rumex conglomeratus | CCTGCTTATACGAAAACT | 5 |
| Rumex obtusifolius |  |  |
| Senecio vulgaris | ATGGTCGTCCTCTAATGGGAT | 8 |
| Setaria verticillata | GTTCCTGGGGAGGCAGA | 7 |
| Sonchus arvensis | GAAGATTTACGAATCCCTA | 4 |
| Sonchus asper |  |  |
| Stachys arvensis | CACATCGAGACCGTTCTT | 10 |
| Taraxacum officinale | CGAATCCCTGTTGCGT | 6 |
| Trifolium pratense | CATGTTTACCTCTATTGTAGG | 3 |
| Trifolium repens | CTACGCCTGGAAGATTT | 3 |
| Veronica agrestis | GATCTTCGACTGGTACAT | 1 |
| Veronica persica | ATCTTCAACCGGTACATGG | 1 |
|  |  |  |
